# Diagnostic value of serum survivin, Ki-67 and thymidine kinase in dogs with nasal cavity disease

**DOI:** 10.3389/fvets.2025.1553551

**Published:** 2025-04-07

**Authors:** Sarah Rösch, Annkathrin Estaller, Gerhard Ulrich Oechtering, Stephan Neumann

**Affiliations:** ^1^Ear, Nose and Throat Unit, Small Animal Department, Leipzig University, Leipzig, Germany; ^2^Institute of Veterinary Medicine, Clinic for Small Animals, University of Goettingen, Goettingen, Germany

**Keywords:** nasal neoplasia, carcinoma, sarcoma, lymphoplasmacytic rhinitis, blood marker, nasal discharge dog

## Abstract

**Background:**

The most common nasal cavity disease (ND) in dogs is the malignant nasal cavity tumor. Prognosis and survival after radiation therapy are reported to correlate with tumor size, and therefore indirectly with the time to diagnosis. Diagnosis of a nasal tumor requires imaging procedures under anesthesia. Thus, diagnostic serum markers are urgently needed for early detection and for therapeutic monitoring.

**Materials and methods:**

This prospective, blinded study included dogs with nasal discharge that completed a comprehensive diagnostic workup for ND. Dogs were evaluated by blood testing and whole-body CT and those with concomitant diseases or with steroid pre-treatment were excluded. Serum survivin, Ki-67, and thymidine kinase 1 (TK1) concentrations were determined, and the survivin-lymphocyte ratio (SLR) calculated. Results were compared between groups of dogs with different NDs and to ten healthy controls.

**Results:**

A total of 55 dogs were included, consisting of 25 with malignant ND (12 sarcomas, 13 carcinomas) and 30 with benign ND (7 benign tumors, 13 dogs with idiopathic rhinitis (IR), 10 others including dogs with dental diseases and sinonasal aspergillosis). Survivin and SLR were significantly increased in dogs with malignant ND and in subgroup comparison in sarcomas compared to controls. In addition, the SLR was significantly increased in carcinomas and IR compared to controls. In dogs with IR, no differences were observed in survivin concentrations or SLR based on microbiological or histopathological findings. Survivin concentrations or SLR in dogs with nasal tumors were not significantly different between T-categories. No significant differences were detected in TK1 concentrations among the groups, nor in Ki-67, except for significantly lower Ki-67 concentrations in benign tumors compared to carcinomas and the group others including, e.g., dental diseases.

**Conclusion:**

Although not diagnostic for ND, increased survivin serum concentrations or SLR can be detected in dogs with malignant nasal tumors and IR. In malignant nasal tumors, survivin and SLR did not correlate with tumor size and therefore may be useful in the detection of even small nasal tumors. Therefore, in dogs with nasal tumors and IR, survivin and SLR could serve as a target for disease monitoring or as therapeutic target.

## Introduction

Malignant nasal tumors are a major cause of chronic nasal discharge in dogs (1, 2). These tumors are diagnosed under anesthesia using computed tomography, rhinoscopy, histopathological and/or cytological examination of tumor biopsies (1, 3). The list of differential diagnoses is long, including benign tumors, oronasal defects, nasal foreign bodies, primary or secondary sinonasal aspergillosis, parasitic diseases and idiopathic rhinitis (IR; diagnosis of exclusion) (1–3). Therefore, novel and specific blood parameters to detect malignant nasal disease at an early stage, monitor response to therapy, and assess the recurrence of nasal tumors after therapy are urgently needed. To date, increased platelet-to-lymphocyte ratio (PLR; sarcomas > carcinomas) and decreased 25(OH)D concentration (sarcomas > carcinomas) can only suggest evidence of a malignant nasal cavity tumor in dogs, indicating a need for investigation into specific serum based markers for disease (4, 5).

Canine IR, a benign inflammation of the nasal mucosa, is the third most common cause of nasal discharge in the literature and is a diagnosis of exclusion (1). The etiology of IR remains unclear. Antibiotics often result in no or only temporary improvement in clinical signs (2, 6). Some dogs can respond to immunomodulatory administration of doxycycline, corticosteroids, cyclosporine or desensitization therapy (2, 7), or other new therapeutic regimens (8), with varying numbers of non-responders in daily clinical practice. It is difficult to objectively assess response to treatment without repeating the cost-intensive examinations under anesthesia for evaluation of the nasal cavity and paranasal sinuses (9).

Survivin, a protein in the ‘inhibitor of apoptosis protein’ family, is highly expressed in tumor and fetal tissues (10). In humans, approximately 79.5% of nasopharyngeal tumors express survivin (11, 12). In dogs, its expression has been detected in several tumors and correlates with tumor malignancy and poor prognosis (12). In a recent study on biopsies of nasal cavity tumors, 100% of carcinomas and 85.7% of sarcomas were survivin positive (13), comparable to another study with 84.9% positivity of nasal carcinomas for survivin (12). The amount of survivin expression in nasal tumor biopsies of carcinomas and sarcomas was not significantly different in regard to tumor size [T-category according to Adams’ proposed modified staging system for canine nasal tumors (14)] (13). In addition to immunohistochemical studies on tumor tissues in dogs, increased survivin serum concentrations have been detected in dogs with cancer (15). However, the correlation between survivin serum concentrations and survivin expression in tumor tissue has only been studied in humans and not in dogs (16). Interestingly, increased survivin expression has also been previously described in mucosa biopsies of humans with chronic rhinosinusitis (CRS) (17).

Ki-67 is only expressed during the G1, S, and G2 phases of the cell cycle, and therefore, is a marker of proliferating cells, and consequently of tumors. Malignant perianal tumors in dogs have significantly higher Ki-67 concentrations than benign ones (18), and in nasal tumors, 28.5% of carcinomas and 17.3% of sarcomas were Ki-67 positive (13). Moreover, Ki-67 concentrations are increased in the serum of dogs with malignant tumors (19).

Thymidine kinase 1 (TK1) is a biomarker of cell proliferation associated with mitotic cell division. Increased extracellular concentrations of TK1 reflect sustained cell proliferation and the number of cells dying during replication releasing TK1 into the bloodstream (20, 21). Therefore, serum TK1 (sTK1) was used to screen for occult canine cancer (22). Serum TK1 expression is increased in dogs with neoplastic diseases, especially those with lymphomas (20).

This study aimed to evaluate the diagnostic utility of the concentrations of serum tumor markers survivin, Ki-67, TK1 and the survivin-lymphocyte ratio (SLR) in a population of dogs with nasal diseases, in which other obvious neoplastic diseases were excluded by clinical examination, basic blood work and whole-body computed tomography. We hypothesized that these serum tumor markers and SLR are considerably increased in dogs with malignant nasal tumors compared to dogs with benign nasal diseases or to dogs with benign nasal tumors.

## Methods

### Study design, animals and ethics approval

This prospective, blinded study was conducted in dogs with nasal discharge presented to the Ear, Nose and Throat Unit of the Small Animal Department at the Faculty of Veterinary Medicine of the Leipzig University for evaluation of nasal discharge. Informed consent was obtained from owners, and the protocol was independently reviewed and approved by the ethics committee at Saxony (animal experiment subject to approval TV 02/18, Saxony, Germany; 08/18–07/20) (4, 5).

All examinations were performed in a standardized manner as previously described (4, 5). Blood samples including blood count (EDTA, blood smear), blood chemistry and electrolytes (heparin plasma and serum) were examined at Laboklin (Bad Kissingen, Germany). Survivin and Ki-67 were measured in the laboratory of the Institute of Veterinary Medicine, University of Goettingen, Germany, and sTK1 was measured at Laboklin (Bad Kissingen, Germany).

In all dogs, a whole-body CT examination with intravenous contrast injection was performed under anesthesia followed by endoscopy of the upper airways including evaluation of the pharynx, larynx, nasopharynx, and trachea (2, 4, 5). Sterile nasal mucosal swabs were taken from deep within the nasal passages and submitted for culture-based microbiological and mycological examination at IDEXX Laboratories (Kornwestheim, Germany) by semi-quantitative evaluation (5). Correlation of microbiology results and serum marker concentration was determined.

Histopathological examination of nasal mucosal biopsies or tissue endoscopically classified as tumor tissue was examined at a specialized institution (Antech Lab Germany GmbH, Tierpathologie Munich, Munich Germany, with Dr. W. von Bomhard, Dipl. ECVP) (4). As described before, the marker serum concentrations were evaluated in correlation to the type of inflammation and the dominant inflammatory cell type (4).

### Nasal disease groups

Dogs were divided into combined groups and subgroups according to the diagnosis of nasal disease: nasal disease (all dogs with ND), malignant nasal tumors (malignant ND) with subgroups of carcinomas (C) and sarcomas (S), benign nasal cavity disease (benign ND) with subgroups of benign nasal tumors (BT; control examination, re-biopsy and repeated histopathological examination confirming benign tumor after 2 months), idiopathic rhinitis (IR; diagnosis of exclusion), and others (O). The group others consisted of dogs with benign diseases other than IR and BT, such as dental diseases or oronasal defects and primary or secondary aspergillosis (4). Dogs with malignant and benign nasal tumors were grouped in regard to tumor size into T-categories 1 to 4 according to Adams’ proposed modified staging system for canine nasal tumors (14). Included dogs were followed up by telephone calls and repeatedly examined during course of disease under anesthesia up to 2 years after the end of the study. As described before, unfortunately some dogs were lost to follow up (5).

Ten normocephalic, healthy dogs served as controls (control group; CG; healthy at the time of examination and 4 weeks later in a physical control examination). As in dogs with nasal disease, additional diseases in healthy control dogs were excluded by means of a clinical examination, blood examination and whole-body CT. Health of the nasal cavity was also evaluated by rhinoscopy, microbiological and mycological examination of a nasal swab and by histopathological examination of nasal mucosa biopsies, as described above.

As described before (4, 5), any dog with nasal disease diagnosed with other neoplasia or systemic diseases, as determined through anamnesis, clinical examination, whole-body CT, and/or blood tests, as well as dogs receiving corticosteroids within 14 days prior to presentation, were excluded to avoid any impact on blood parameters and serum markers.

### Measurements of survivin, Ki-67, and sTK1, and calculation of the SLR

Survivin and Ki-67 serum concentrations were measured using commercially available immunoassay kits (E08S0218, canine survivin ELISA kit, competitive enzyme immunoassay technique; BlueGene, Shanghai, China; and MBS089640 canine Ki-67 protein ELISA kit, quantitative sandwich ELISA; MyBioSource, San Diego, CA, USA), with non-diluted serum in duplicate, adhering to the manufacturer’s instructions. Briefly, serum samples were added to a pre-coated 96-well microtiter plate, which was then covered and incubated for 1 h at 37°C. This was followed by five washes to remove all unbound antibodies. A solution containing an enzyme-conjugated detection antibody that binds specifically to the antigen was added, followed by another incubation with coverage for approximately 15 min at 37°C. The substrate forms a colored solution when catalyzed by the addition of an acid solution, stopping the antigen–antibody reaction. The absorbance was measured spectrophotometrically at 450 nm using a Magellan optical emission spectrometer. A standard curve was constructed using CurveExpert Professional^1^ to calculate survivin and Ki-67 serum concentrations in pg/mL. The detection range of the ELISA in serum samples, as per the manufacturer’s instructions, was 25–1.000 pg/mL and 0.625–20.0 ng/mL for survivin and Ki-67, respectively. The manufacturer reported mean intra-assay and inter-assay coefficients of variation of 4.4 and 6.6%, respectively, for survivin, and mean intra-assay and inter-assay coefficients of less than 15% for Ki-67.

The concentrations of sTK1 were determined using an indirect, competitive chemiluminescence immunoassay (CLIA; Liaison XL device; Diasorin, Saluggia, Italy) validated and used in dogs (23, 24) with a minimum detectable activity of 0.2 U/L (21). Serum TK1 concentrations were normal at concentrations ≤1.9 U/L, increased at 2–8.9 U/L, and markedly increased at ≥9 U/L (21). In contrast to the survivin and Ki-67 studies conducted in Göttingen, the sTK1 study was conducted in a blinded fashion at Laboklin (Bad Kissingen, Germany).

The survivin to lymphocyte ratio (SLR) was calculated using the concentrations of serum survivin and absolute lymphocyte count from EDTA blood, as previously reported (4). Additionally, correlations among the three serum tumor markers and previous reported markers were calculated (4, 5).

In one dog with IR, survivin and Ki-67 serum concentrations could not be determined, and in two dogs with carcinoma and two dogs with sarcoma, sTK1 concentrations could not be determined (sTK1 concentrations in 51/55 dogs available).

### Statistics

Statistical analyses were performed using GraphPad Prism (v9 GraphPad Software, La Jolla, CA, USA) as previously reported (4, 5). The values were tested for normal distribution using the D’Agostino & Pearson normality test and Shapiro–Wilk normality test. The data on survivin, Ki-67, serum thymidine kinase, and SLR were not normally distributed. Comparisons among non-parametric group data were performed using the Kruskal–Wallis test and Dunn’s multiple comparisons test. Results are expressed as median values with interquartile ranges (IQR). For the post-hoc comparison of two non-parametric groups, the Mann–Whitney test (two-tailed) was used (25). Statistical significance was set at *p* < 0.05. Spearman non-parametric correlation (Spearman correlation coefficient = rs) was used to evaluate the correlation between serum tumor markers. Receiver operating characteristic (ROC) curves of survivin and SLR were determined to evaluate the sensitivity and specificity for diagnosing malignant tumors and/or IR. The ROC curves showed sensitivity versus specificity such that the area under the curve (AUC) varied from 0.5–1.0, with higher values indicating increased discrimination.

## Results

### Nasal disease groups

**Figure 1:**
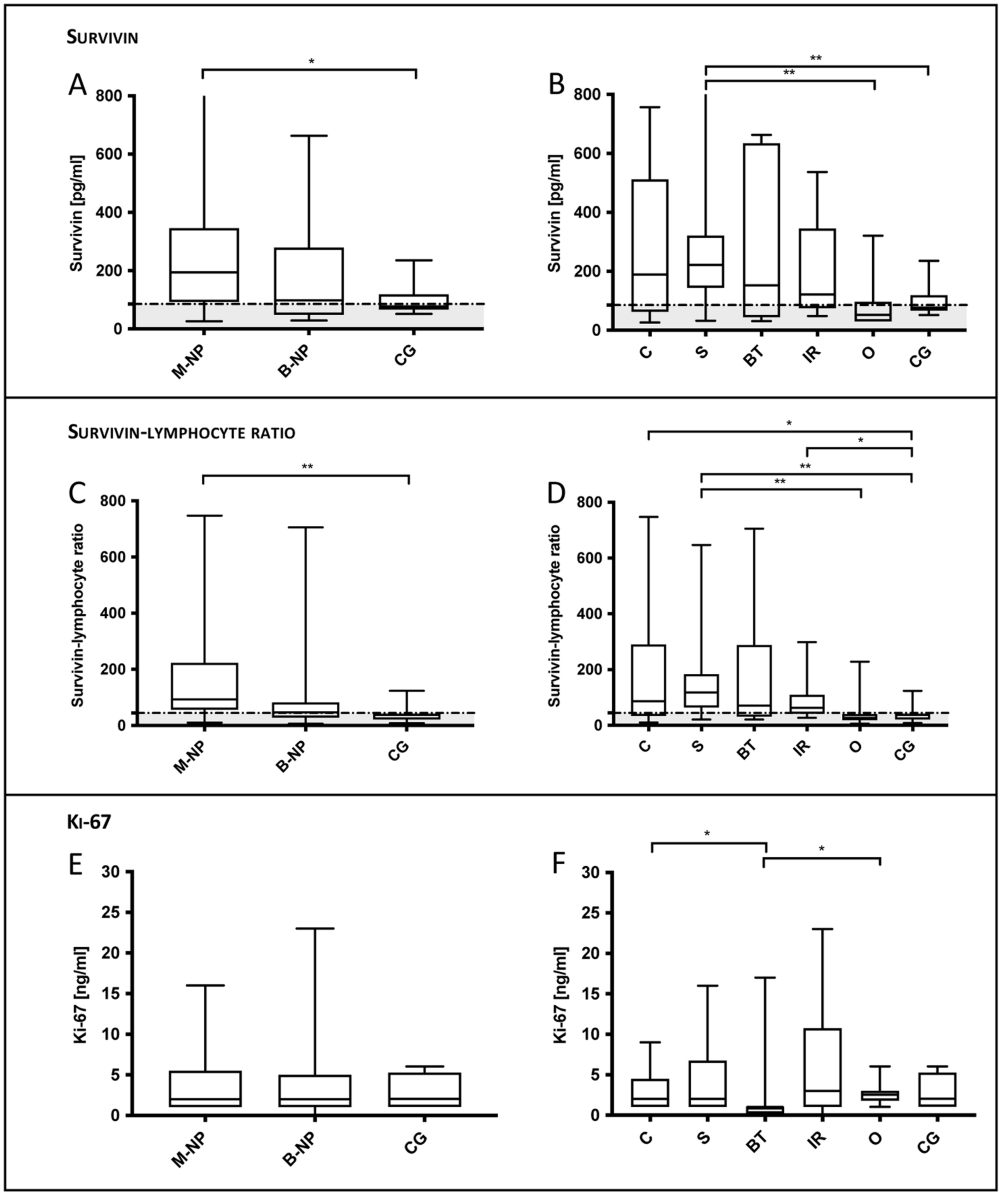
Serum survivin, survivin-lymphocyte ratio (SLR) and serum Ki-67 in dogs with chronic nasal discharge. **(A)** Compared to controls (CG), survivin was significantly increased in dogs with malignant ND (*p* = 0.028) and **(B)** in subgroup comparison significantly increased in dogs with sarcomas (S; *p* = 0.009). Additionally, survivin concentrations of dogs with S were significantly increased compared to dogs of the group others (O; *p* = 0.003). **(C)** SLR in malignant ND was significantly increased compared to controls (*p* = 0.002), **(D)** as well as in subgroup comparison in dogs with S (*p* = 0.001), carcinomas (C; *p* = 0.030) and idiopathic rhinitis (IR; *p* = 0.014). Additionally, the SLR in S was significantly increased compared to that of O (*p* = 0.001). **(E, F)** Only Ki-67 concentrations in dogs with C (*p* = 0.040) and O (*p* = 0.026) were significantly increased compared to concentrations in benign tumors. Data are shown in box and whisker plots. Upper and lower boxes represent the 25th and 75th percentiles (lower whiskers = minimum, upper whiskers = maximum values) and the line represents the median. In the graphs, the in the present study calculated cut-off values (88.5 pg/mL for survivin and 44.55 for SLR) are marked by a dotted horizontal line.

55 of 72 dogs were included in the study. 25/55 dogs (45%) were assigned to the malignant ND group with 13 dogs with carcinomas and 12 dogs with nasal sarcomas. Thirty of fifty-five dogs (55%) were diagnosed with benign inflammatory nasal cavity diseases. Seven of 30 dogs (23%) were diagnosed with a benign nasal neoplasia, 13/30 dogs (43%) with IR and 10/30 dogs (34%) with other inflammatory diseases (Figure 1). Ten healthy dogs served as control animals. The median values of the serum tumor markers are shown in Table 1.

In the IR group, the biomarkers and the SLR were not significantly different between dogs with different nasal mucosal inflammation types or in regard of a positive culture-based microbiological examination (*data not shown*).

The size of the nasal cavity tumours in the CT-examination was assessed as T-category 1 in 6 dogs (3 carcinomas, 2 sarcomas and one benign tumour), as T-category 2 in 3 dogs (2 carcinomas and one sarcoma), as T-category 3 in 12 dogs (1 carcinoma, 5 sarcomas, 6 benign tumours) and as T-category 4 with lysis of the cribriform plate in 10 dogs (7 carcinomas and 3 sarcomas). Median duration of clinical signs in included dogs with ND was 5 months (IQR: 2–10).

**Table 1:** Serum marker survivin, Ki-67 and serum thymidine kinase in dogs with nasal cavity diseases (ND) and in controls.

		Dogs with nasal cavity disease and nasal discharge					Controls
	Unit and Reference interval for sTK1	Carcinomas 13/55 dogs	Sarcomas 12/55 dogs	Benign Tumors 7/55 dogs	Idiopathic Rhinitis 13/55 dogs	Others 10/55 dogs	Healthy 10 dogs
Survivin	pg/mL	189 [IQR: 62.5–512]	221.5 [IQR: 143.5–321.8]	152 [IQR: 44–635]	121.5 [IQR: 74.3–345.5] *in 12/13 dogs*	51.5 [IQR: 29–96]	76.5 [IQR: 65.8–118]
Survivin-lymphocyte ratio		86.4 [IQR: 34–290.2]	118.4 [IQR: 63.4–183.8]	71.3 [IQR:31–288.3]	63.2 [IQR:42–109.7] *in 12/13 dogs*	26.6 [IQR:18.1–40.8]	38.7 [IQR:21.1–41.9]
Ki-67	ng/mL	2 [IQR: 1–4.5]	2 [IQR: 1–6.8]	1 [IQR: 0.2–1]	3 [IQR: 1–10.8] *in 12/13 dogs*	2.5 [IQR: 1.8–3]	2 [IQR: 1–5.3]
Serum thymidine kinase	≤ 1.9 U/L	2.9 [IQR: 0.7–6.9]	5.4 [IQR: 0.5–17.9] *in 11/13 dogs*	2.1 [IQR: 0.7–4.9] *in 10/12 dogs*	5 [IQR: 1.6–6.2]	2.4 [IQR: 1.2–6.2]	2.7 [IQR: 1.5–4.3]

Seventeen of the seventy-two dogs initially recruited were excluded due to one or more of the following recognized diseases, or because they were treated with steroids (*n* = 5) for one of the following underlying chronic diseases (5). As reported before (5), systemic diseases included polyarthritis or osteoarthritis (*n* = 5), pulmonary fibrosis, infectious/bacterial pneumonia (*n* = 2, one dog was diagnosed based on bronchoalveolar lavage results and another based on the clinical signs of fever and pneumonia responsive to antibiotics), mast cell tumor, tumor ventral to the urinary bladder, leishmaniasis (*n* = 2), structural abnormalities of the liver parenchyma (*n* = 3), hepatic shunt, chronic pancreatitis, acute gastroenteritis, inflammatory bowel disease, and protein-losing enteropathy (5).

### Measurements of survivin and calculation of the SLR

In dogs with malignant ND (median 194 pg/mL, IQR: 92–346 pg/mL) survivin serum concentrations were significantly increased compared to controls (median 76.5 pg/mL, IQR: 65.8–118 pg/mL; *p* = 0.028; Figure 1; Table 1). Compared to controls, survivin concentrations were significantly increased in dogs with sarcoma (median 221.5 pg/mL, IQR: 143.5–321.8 pg/mL; *p* = 0.009). Additionally, the survivin values in dogs with sarcomas were significantly increased than those in dogs in the group others including dogs with dental diseases or sinonasal aspergillosis (median 51.5 pg/mL, IQR: 29–96 pg/mL; *p* = 0.003).

SLR was significantly increased in dogs with malignant ND (median 92.8, IQR: 56–223.5) compared to controls (median 38.7, IQR: 21.1–41.9; *p* = 0.002; Figure 1). In the subgroup comparison, the SLR in dogs with sarcoma (median 118.4, IQR: 63.4–183.8) was significantly increased compared to controls (*p* = 0.001) and to dogs in the group others (median 26.6, IQR: 18.1–40.8; *p* = 0.001). Furthermore, compared to controls, the SLR in dogs with carcinoma (median 86.4, IQR: 34–290.2; *p* = 0.030) and IR (median 63.2, IQR: 42–109.7; *p* = 0.001) were significantly increased.

ROC curve analyses were performed for different group combinations (Table 2) to distinguish healthy dogs and dogs from the group others from the two groups with increased survivin concentrations (nasal tumors and IR). Comparable cut-off values were determined for the different group compositions (Table 2). Combining dogs with malignant tumors and IR, the ROC curve analysis showed an AUC of 0.77 (95% CI, 0.64–0.89) for survivin and an AUC of 0.83 (95% CI, 0.71–0.94) for SLR. Taken both groups together, an SLR > 44.55 showed a sensitivity of 81.08% and a specificity of 90%, while a survivin value >85.5 showed a sensitivity of 78.38% and a specificity of 70% (Table 2).

**Table 2:** Evaluation of cut-off values for survivin serum concentrations and SLR in relation to different group combinations and comparisons.

	AUC	Value	Sensitivity %	95% CI	Specificity %	95% CI	Likelihood ratio
SLR							
Sarcoma vs. O/C	0.88 (95% CI: 0.75–1)	> 47.40	91.67	64.61% to 99.57%	90.00	69.90% to 98.22%	9.167
Malignant tumor vs. O/C	0.83 (95% CI: 0.7–0.96)	> 44.55	84.00	65.35% to 93.60%	90.00	69.90% to 98.22%	8.400
**Malignant tumor and IR vs. O/C**	**0.83** (95% CI: 0.71–0.94)	**> 44.55**	**81.08**	**65.80% to 90.52%**	**90.00**	**69.90% to 98.22%**	**8.108**
Malignant and benign tumor and IR vs. O/C	0.82 (95% CI: 0.71–0.93)	> 39.95	81.82	68.04% to 90.49%	75.00	53.13% to 88.81%	3.273
IR vs. O/C	0.83 (95% CI: 0.68–0.98)	> 45.60	75.00	46.77% to 91.11%	90.00	69.90% to 98.22%	7.500
Survivin							
Sarcoma vs. O/C	0.85 (95% CI: 0.69–0.99)	> 140.0	83.33	55.20% to 97.04%	85.00	63.96% to 94.76%	5.556
Malignant tumor vs. O/C	0.78 (95% CI: 0.64–0.92)	> 94.00	76.00	56.57% to 88.50%	80.00	58.40% to 91.93%	3.800
**Malignant tumor and IR vs. O/C**	**0.77** (95% CI: 0.64–0.89)	**> 85.50**	**78.38**	**62.80% to 88.61%**	**70.00**	**48.10% to 85.45%**	**2.613**
Malignant and benign tumor and IR vs. O/C	0.76 (95% CI: 0.64–0.88)	> 92.50	75.00	60.56% to 85.43%	75.00	53.13% to 88.81%	3.000
IR vs. O/C	0.75 (95% CI: 0.57–0.93)	> 92.50	75.00	46.77% to 91.11%	75.00	53.13% to 88.81%	3.000

For both markers, a cut-off was chosen to differentiate between malignant tumors (carcinomas and sarcomas) as well as IR. The in the diagrams chosen group and cut-off values are highlighted in bold. These cut-off values are represented in the figures of the present study (for survivin and SLR) by a horizontal dotted line.

**Figure 2:**
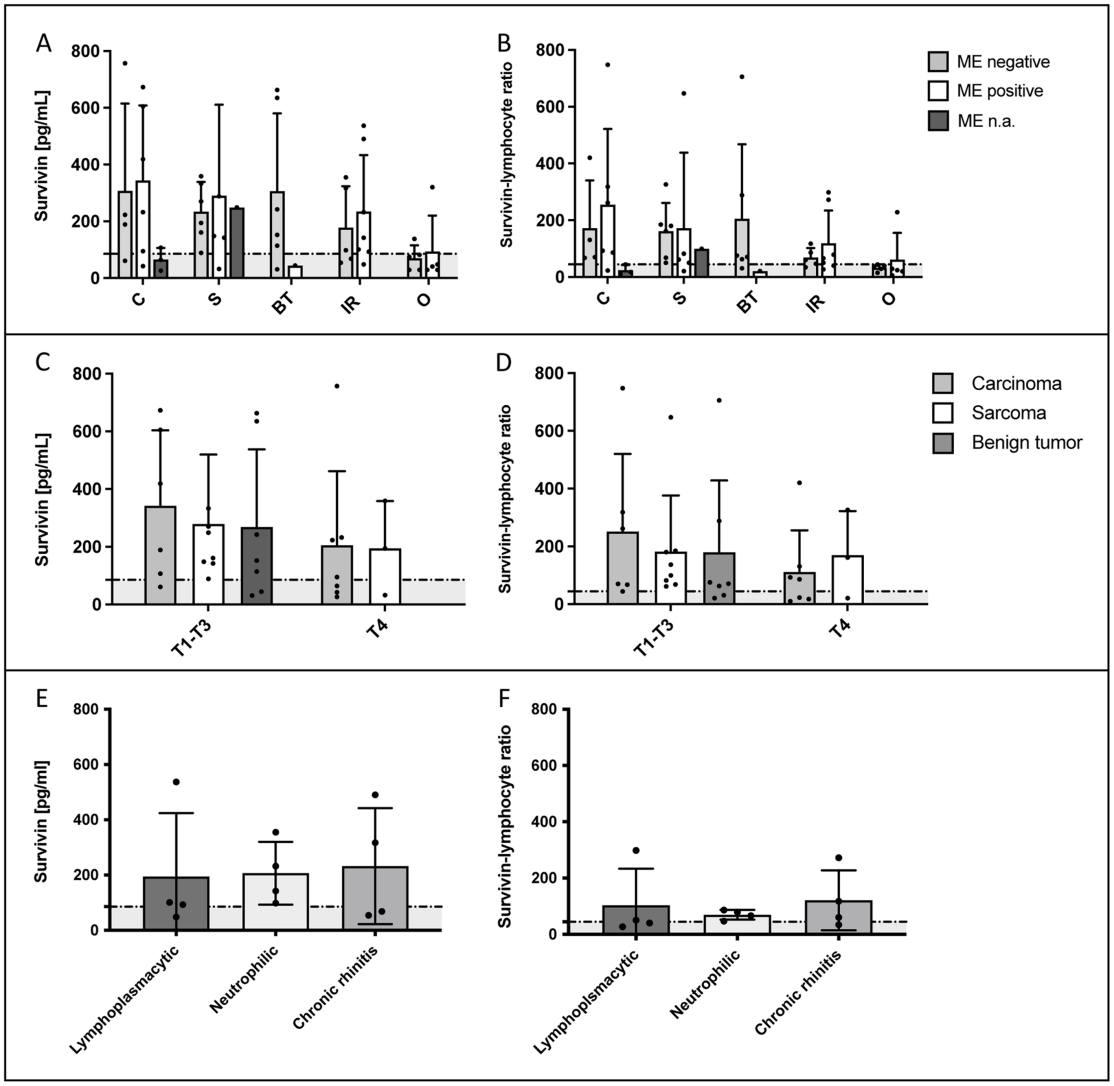
Evaluation of the influence of various factors on survivin serum concentrations in dogs with idiopathic rhinitis (IR) or malignant nasal tumors. **(A,B)** show the survivin concentrations or SLR in the different groups in relation to a positive or negative result of the microbiological examination (ME) of a nasal swab. No significant differences in sense of influence of bacterial colonization on marker concentrations were detected. n.a. = ME was not available in 4 dogs with nasal tumor due to financial constraints of the owners (*n* = 3) or because it was performed at the regular veterinarian (*n* = 1) before presentation. **(C,D)** Additionally, no statistically significant difference in survivin concentrations or SLR was detected in the different T-categories [T-category according to Adams et al. (14)] with T4 indicating cribriform plate lysis. Due to the low number of dogs with T1-T3 category in different tumor groups, these categories were grouped together (6 carcinomas, 8 sarcomas, and 7 benign tumors) against T4 (7 carcinomas and 3 sarcomas). **(E,F)** In dogs with IR, the result of the histopathological examination in regard of the predominant inflammatory cell type is shown. No statistically significant differences in serum survivin concentrations or SLR were detected. C = carcinoma, S = sarcoma, BT = benign tumor, IR = idiopathic rhinitis, O = others. In the graphs, the in the present study calculated cut-off values (88.5 pg/mL for survivin and 44.55 for SLR) are marked by a dotted horizontal line.

**Figure 3:**
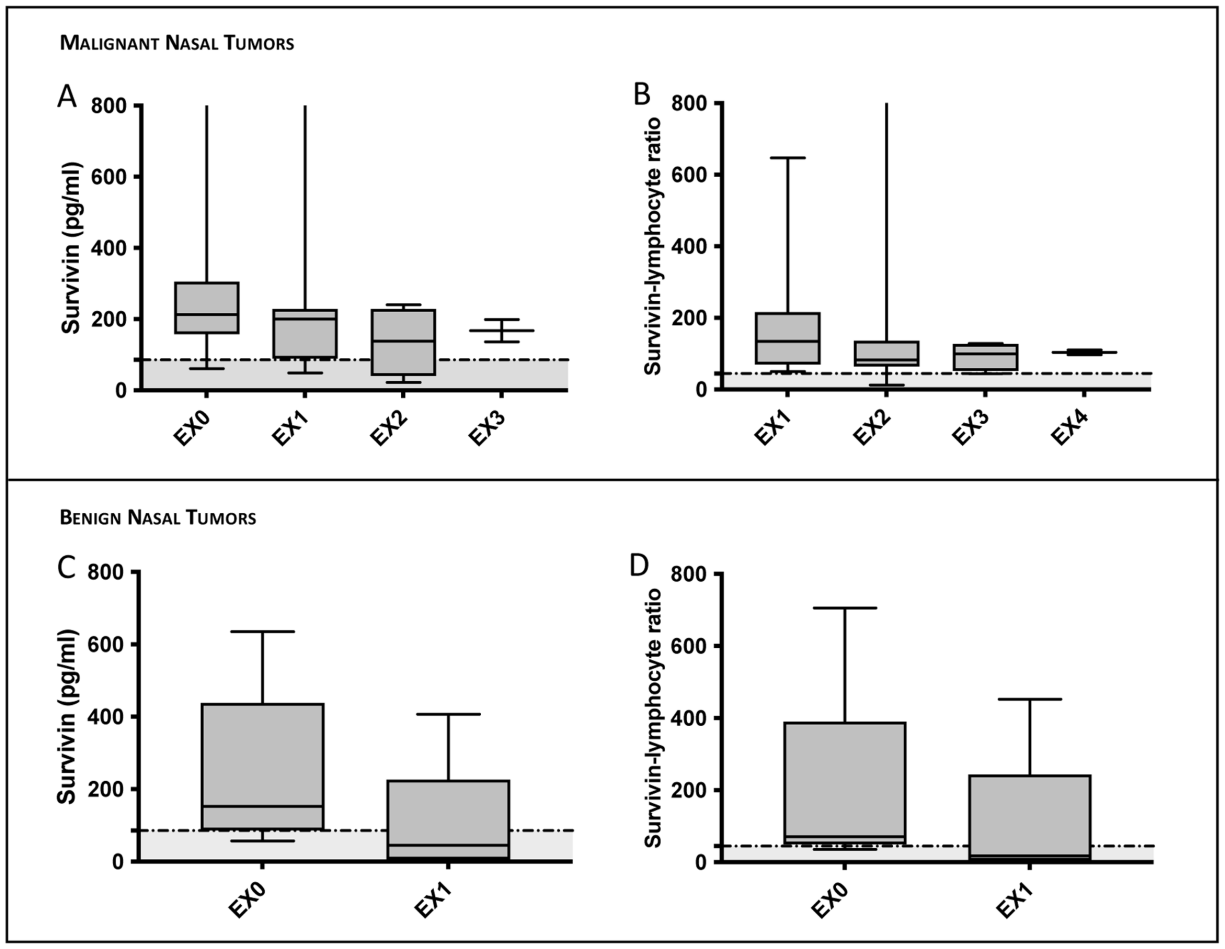
Survivin serum concentrations and survivin-lymphocyte ratio (SLR) in dogs detected in control examinations (EX). The dogs had not received any steroids between examinations. In panel **(A–D)** survivin concentrations and SLR 6–8 weeks after diagnosis and palliative endoscopic interventional cytoreduction through the nasal entrance are illustrated in malignant **(A,B)** and benign **(C,D)** nasal tumors. In some dogs, re-examinations were performed on owner’s request. In panel **(A,B)** 7 sarcomas and 3 carcinomas were evaluated (with only 4 concentrations in EX2 and 2 concentrations in EX3 available). In panel **(C,D)** values of 5 benign tumors are illustrated at two different time points, 6–8 weeks apart. Despite tumor volume reduction, survivin concentrations and SLR values did not show a statistically significant decrease. **(C,D)** However, in dogs with benign tumors, concentrations fell below the cut-off values set in the present study. In the graphs illustrating survivin concentrations and SLR respectively, the previously calculated cut-off values (88.5 pg/mL for survivin and 44.55 for SLR) are marked by a dotted horizontal line. Data are shown in box and whisker plots. Upper and lower boxes represent the 25th and 75th percentiles (lower whiskers = minimum, upper whiskers = maximum values) and the line represents the median.

In dogs with IR, as in dogs of other groups, no statistically significant differences were observed in survivin concentrations or SLR in regard of the result of the culture-based microbiological examination (Figures 2A,B), or the predominant cell type in the inflammation of the nasal mucosa (Figures 2E,F). In dogs with nasal tumors, tumor size did not affect survivin concentrations or SLR (Figures 2C,D). Therefore, it is not surprising that no significant decrease in survivin concentrations or SLR were detected during rechecks of dogs with nasal tumors treated with palliative cytoreduction of the tumor by endoscopic intervention (Figure 3).

### Evaluation of survivin and of the SLR in excluded dogs with ND with another detected disease and/or steroid pre-treatment

**Figure 4:**
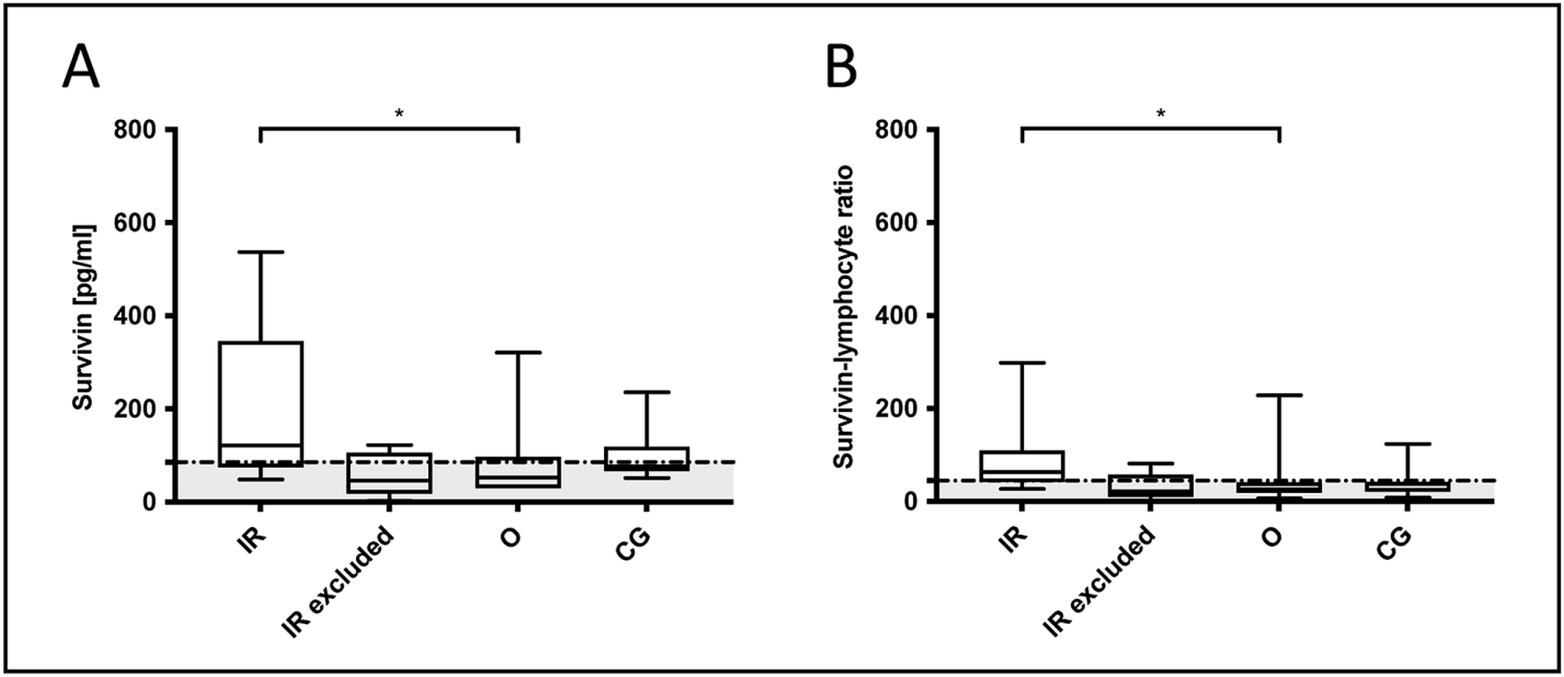
Comparison of **(A)** survivin concentrations and **(B)** survivin-lymphocyte ratio (SLR) in included dogs of the groups others, the control group and dogs with idiopathic rhinitis (IR) versus excluded IR dogs. If evaluating only dogs without a detectable nasal mass, including the control dogs, survivin concentrations and SLR were significantly increased in dogs with IR, in contrast to the other group with dental diseases or sinonasal aspergillosis (Kruskal-Wallis test: survivin *p* = 0.049 and SLR *p* = 0.029). In excluded dogs, survivin and SLR were suggested to be decreased due to steroid pre-treatment or pneumonia, as in human medicine both variables were reported to negatively influence survivin values. In the graphs, the in the present study calculated cut-off values (88.5 pg/mL for survivin and 44.55 for SLR) are marked by a dotted horizontal line. Data are shown in box and whisker plots. Upper and lower boxes represent the 25th and 75th percentiles (lower whiskers = minimum, upper whiskers = maximum values) and the line represents the median.

Of the 17 excluded dogs, 5 were excluded due to cortisone administration, and these showed low survivin serum concentrations, with a median of 57 pg/mL [IQR: 18.5–91 pg/mL]. Of these 5 dogs, two would have belonged to the IR group, with survivin values of 90 pg/mL and 2 pg/mL. Additionally, 2 out of the 5 excluded IR dogs had pneumonia, and their survivin concentrations were also low (46 pg/mL and 33 pg/mL). In contrast to these 4 dogs with lung pathology or steroid pre-treatment, one dog with IR, which was excluded due to a detected structural liver change and pancreatitis, had an increased survivin concentration of 122 pg/mL, what is comparable to the other IR dogs (Figure 4).

### Measurements Ki-67 and sTK1

Ki-67 was not statistically significantly different between the controls (Table 1; median 2 ng/mL, IQR: 1–5.3 ng/mL) and malignant ND (median 2 ng/mL, IQR: 1–5.5 ng/mL), or benign ND (median 2 ng/mL, IQR: 1–5 ng/mL). However, Ki-67 expression in dogs with benign tumors (median 1 ng/mL, IQR: 0.2–1 ng/mL; *p* = 0.04) was statistically significantly decreased compared to dogs with carcinoma (median 2 ng/mL, IQR: 1–4.5 ng/mL) and compared to dogs of the group others including dental diseases or sinonasal aspergillosis (median 2.5 ng/mL, IQR: 1.8–3 ng/mL; *p* = 0.026).

The median sTK1 concentrations (Table 1; reference interval ≤ 1.9 U/L) were above the reference interval in controls (median 2.3 U/L; IQR: 1.7–3.4 U/L), malignant ND (median 2.9 U/L; IQR: 0.7–6.9 U/L) and benign ND (median 2.8 U/L; IQR: 1.5–6.2 U/L). The concentrations in the various groups were not statistically significantly different (*data not shown*).

### Correlation among the three serum tumor markers in dogs with ND

No significant correlation was found among serum survivin, Ki-67, and sTK1 concentrations in dogs with ND. However, a positive correlation was observed between sTK1 and haptoglobin, an inflammatory marker, which has been described in another study on this patient population (Spearman *r* = 0.4138 with *p* = 0.003) (5).

## Discussion

In the present study, the serum tumor markers survivin, Ki-67, and TK1 as well as a calculated SLR were investigated in dogs with different ND. Even if all serum tumor markers and the SLR were not diagnostic for ND, the present study shows, that serum survivin and/or SLR were significantly increased in nasal sarcomas and/or carcinomas and IR in comparison to healthy controls. We emphasize that survivin may be a potential future marker that requires further investigations, as markers for treatment monitoring are lacking in malignant nasal tumors and IR. Both diseases are the most common causes of chronic nasal discharge in dogs, emphasizing the need for a suitable marker. Disease relapses still need to be diagnosed by comprehensive diagnostics in anesthesia. In addition, new therapeutic treatment strategies for dogs with IR are needed. Therefore, survivin inhibitors (26) could be investigated in further studies. Our results are consistent with human studies that have found increased survivin serum concentrations in humans with various malignant tumors, e.g., nasopharyngeal tumors (12, 13), and have described increased survivin expression in the mucosa of humans with chronic rhinosinusitis (CRS) (17).

In a previous study conducted by the authors on biopsies of malignant nasal tumors in a different canine population, survivin was already detected in a high number of both types of malignant nasal tumors (13). Interestingly, in contrast to the present study in which higher survivin serum concentrations were detected in sarcomas than in carcinomas, in the cited study, a higher percentage of biopsies from carcinomas were positive for survivin (100% of carcinomas) as opposed to nasal sarcomas at 85.7% (13). One reason for this could be a different correlation between tissue expression and survivin serum concentration in different tumor types. The correlation between tissue expression and serum concentration has so far only been investigated in humans (16). It is known from studies in human medicine that despite high survivin expression in tumor tissue, the concentration of survivin in the serum can be lower (27).

However, the results of the present study, which show higher survivin serum concentrations in sarcomas than in carcinomas, are consistent with a previous study that found higher survivin serum concentrations in dogs with different types of sarcomas than in dogs with carcinomas (28). It is important to note that in contrast to the present study’s survivin concentrations of 189 pg/mL [IQR: 62.5–512 pg/mL] in nasal carcinomas and 221.5 pg/mL [IQR: 143.5–321.8 pg/mL] in nasal sarcomas, higher maximum survivin concentrations were observed in dogs with malignant tumors in the cited study, such as, 104 pg/mL [IQR: 25–1,113 pg/mL] in dogs with adenocarcinomas of the mammary glands, 45 pg/mL [IQR: 10–658 pg/mL] in squamous cell carcinomas, and 93 pg/mL [IQR: 18–5,960 pg/mL] in dogs with soft-tissue sarcomas (28).

In the present study, not significantly different survivin concentrations were found in dogs with different tumor sizes [T-categories according to Adams et al. (14)]. This is consistent with our previous study on tissue biopsies of nasal carcinomas and sarcomas (13). It also correlates with the finding, that survivin did not decrease significantly in repeated measurements at control examinations despite reduction of tumor size with palliative endoscopic interventional cytoreduction. Due to studies currently being completed, this method and the results cannot be discussed further. However, it should be noted that increased survivin concentrations can remain increased in the serum even with small residual amounts of malignant tumor tissue in the nasal cavity, making survivin an ideal tumor marker in need of further investigations.

In the present study, SLR with inclusion of lymphocyte values from a previous study (4) made the differences between the groups even clearer when only the absolute survivin value was considered. These results are consistent with those of a previous study (29). Therefore, in the present study, evaluation of SLR revealed not only a significant difference between dogs with sarcomas and the control group or the other group, but also a significant difference between dogs with carcinomas and IR compared to the control group.

In human medicine patients with CRS can be differentiated into CRS with and without nasal polyposis (29). In addition to increased survivin concentrations in CRS, Fruth et al. showed significantly increased tissue expression of survivin in polyp tissues from patients with CRS (30). Since, in contrast to humans, the occurrence of benign nasal polyps in chronic idiopathic rhinitis is rare in dogs (8), and the etiopathogenesis of polyps is unclear (31), dogs with benign nasal tumors were considered separately in the group of benign nasal tumors in the present study. Dogs with polyps and benign nasal hamartomas were included. Repeated biopsies were taken to avoid misdiagnosis as there are only few case reports of benign nasal hamartomas in dogs (32), and benign tumors are generally considered less common than malignant tumors (2). Similar to canine nasal polyps, the etiology of canine hamartomas is unclear. In humans, Nguyen et al. described a frequent co-occurrence of hamartomas with human nasal polyps, suggesting a consequence of chronic inflammation in both pathologies in humans (33). In the present study, possibly due to the small number of dogs in this group, and despite some higher survivin concentrations in some dogs with benign tumors, no statistically significant differences to malignant tumors, to the other group or especially IR could be detected. Therefore, the marker survivin or the SLR (a) do not provide clear evidence for a possible common etiopathogenesis between benign tumors and IR and (b) cannot be used to differentiate between benign and malignant nasal tumors or dogs with IR.

Since bacterial colonization of the dog’s nasal mucosa may have influenced the release of markers into the blood and the measurement, because cytokines in the nasal mucosa have been described to be influenced by toxins such as those released by intracellular *Staphylococcus aureus* (34), we investigated the difference in markers in dogs with positive or negative culture-based microbiological examination. Comparable to the previous study (5), the results of this examination had no significant influence on the serum marker concentrations of TKI, Ki-67 and survivin in the dogs of the different groups.

No serum marker for therapy monitoring of IR or nasal tumors has been established in previous research. In the present study, we discuss the two most promising markers identified in our previous and present research in Table 3: canine C-reactive protein (CRP) (5) and survivin or SLR. CRP showed statistically significantly increased concentrations in all dogs with ND compared to controls. However, in all groups of dogs with ND it was unfortunately only slightly above the reference interval and as an acute phase protein, it could be easily influenced by other diseases. In dogs with IR, CRP was also not significantly different in dogs with positive or negative microbiological examination of a nasal cavity swab, so unfortunately it remains unclear whether a secondary bacterial infection can be present in these dogs, or if there is need for therapeutic intervention, as it is evaluated in human medicine (35). Furthermore, CRP was not significantly elevated and thus not diagnostically useful in dogs with nasal cavity tumors with cribriform plate lysis (5). These conditions therefore do not make CRP a good marker for monitoring nasal cavity disease. However, it is advantageous to recognize that in dogs with diseases with high CRP values, such as steroid-responsive meningitis-arteritis or immune-mediated polyarthritis (36), and nasal discharge, high CRP values are probably not influenced by the nasal cavity pathology (even in dogs with T-category 4 of a nasal tumor). Additionally, CRP is obviously useful in dogs with IR, as it can indicate pneumonia (37, 38), and dogs with IR are suggested to be predisposed to secondary pneumonia (39).

**Table 3:** Conclusions from the results of the present study on serum survivin and SLR compared to a previous studies on canine C-reactive protein (CRP) (5).

Acute phase protein canine C-reactive protein (CRP)	Not diagnostic for diseases of the nasal cavity, as median values were slightly increased in every nasal cavity disease group. Unsuitable for monitoring IR or nasal tumors, because CRP serum concentrations are within or only slightly above the reference interval. No statistically significant difference in CRP serum concentrations were found with respect to the histopathological type of mucosa inflammation or microbiological test results in IR dogs. CRP does not show a correlation with tumor size or destruction of the cribriform plate. CRP is easily influenced by other diseases, but obviously not steroid pretreatment. Diseases such as steroid-responsive meningitis-arteritis or immune-mediated polyarthritis (36) could be monitored by CRP concentrations even in the presence of nasal disease. Particularly important for dogs with IR: CRP is increased in (secondary) bacterial pneumonia (37, 38) [= a possible complication in dogs with IR (39)].
Survivin and SLR	SLR offers better diagnostic value (higher AUC) for group differentiation, making it the preferred marker for canine patient evaluation (even if this would make a hematological examination including lymphocyte count necessary). Survivin and SLR are not diagnostic for nasal cavity disease, as they are increased in dogs with nasal tumors and IR. Therefore, they could be helpful in differentiating between IR and other benign diseases, but not between IR and nasal tumors. No statistically significant difference in survivin concentration or SLR were found with respect to the histopathological type of mucosa inflammation or microbiological test results in IR dogs. Survivin/SLR do not show a correlation with tumor size or destruction of the cribriform plate. Therefore, survivin/SLR could be useful for monitoring IR and tumor disease and further investigations are needed. As described in humans (41), survivin could be decreased in dogs with pneumonia (a common complication in dogs with IR), making this marker less ideal. Further studies are needed to explore the role of survivin in dogs with pneumonia, as this could affect the marker’s utility in dogs with IR. Survivin serum concentrations may be decreased in patients with prior steroid treatment (40), as seen in the present study with dogs pre-treated with prednisolone. This possible influence needs further investigation and has to be considered when interpreting survivin concentrations.

In IR dogs, survivin may be beneficial for disease diagnosis and monitoring in several ways and needs further investigations. Survivin may help to differentiate dogs with IR from non-tumor-diseased dogs with dental disease or aspergillosis. However, the number of dogs with primary aspergillosis in the present study was small and needs further investigation. Obviously, and as reported from human medicine, survivin concentrations can be decreased when steroids are administered (40) or in case of pneumonia (41), which should be considered in the evaluation of the values.

Serum Ki-67, a nuclear protein that protects proliferating cells from damage, is increased in malignant tumors (12) and in sera of dogs with tumors (19). In a previous study (13), 28.5% of 29 biopsies of malignant nasal tumors were positive for Ki-67. In the present study, serum Ki-67 was measurable in all groups of dogs, including malignant NDs, IR, and controls, with comparable median Ki-67 values. Strikingly, Ki-67 serum concentrations were significantly lower in dogs with benign NDs. This could be due to the lower number of animals or variations in the pre-analytical results. However, Ki-67 serum concentrations in dogs with malignant NDs in the present study (2 ng/mL with an IQR of 1–4.5 ng/mL for carcinomas and 1–6.8 ng/mL for sarcomas) were not as high as reported for dogs with other neoplastic diseases with a median serum Ki-67 concentration of 243 pg/mL (IQR: 0–7,500 pg/mL) (19). The discrepancy in these results could be attributed to the use of different immunoassay kits (19). There was no difference in the concentration of Ki-67 (or other serum markers) detected with respect to breed or gender of the included dogs (*data not shown*).

Serum thymidine kinase 1 is a cytoplasmic enzyme whose expression correlates with DNA synthesis and cell proliferation (42). Significantly higher concentrations of sTK1 were observed in dogs with lymphoma than in dogs with inflammatory diseases or control dogs (20). In addition, sTK1 is an indicator of response to treatment (20). However, in the present study, no significant differences in sTK1 concentrations were observed among the different groups, suggesting that sTK1 is not a useful marker for detecting malignant nasal tumors. This contrasts with other studies suggesting sTKI as a suitable marker for detection of occult cancer (43).

Limitations of the present study include the low number of dogs of benign tumors or aspergillosis. The control group consisted nearby only of dogs of one breed (beagles), what could have a breed-related influence on serum tumor markers. The dogs with nasal discharge were recruited at a tertiary institution and selection bias cannot be excluded.

## Conclusion

Even if not diagnostic for ND, increased survivin serum concentrations or SLR can be detected in dogs with malignant nasal tumors or IR. Therefore, in both diseases, survivin may act as a target for disease monitoring and in IR dogs potentially as a therapeutic target. Despite description as a marker for occult neoplastic diseases, TK1 is not diagnostic for malignant nasal tumors in dogs.

## Data Availability

The original contributions presented in the study are included in the article/supplementary material, further inquiries can be directed to the corresponding authors.

## Footnotes

^1^Hyams Development, https://www.curveexpert.net/
